# Operationalising place for land system science

**DOI:** 10.1007/s11625-020-00827-5

**Published:** 2020-06-20

**Authors:** Michal Switalski, Adrienne Grêt-Regamey

**Affiliations:** Planning of Landscape and Urban Systems, ETH Zurich, HIL Stefano-Franscini-Platz 5, 8093 Zurich, Switzerland

**Keywords:** Place, Place-making, Land system science, Design, Peri-urbanisation, Land change modelling

## Abstract

The following paper introduces the concept of place for land system science to better understand how the transformation of place, as place-making, can be operationalised. The aim is to operationalise place with the motivation that a deeper understanding of people–place interactions can advance knowledge of land systems towards practicable solutions to current sustainability challenges. An overview of place studies spanning a wide range of research disciplines is presented to form a clear and concise theoretical foundation, necessary when operationalising place beyond its traditional research domains and applications. The limitations and potential of place in the context of land systems science are then explored through examples and the importance of operationalising place as both a product and process is demonstrated. Place and place-making are presented as a conceptual model, which allows for expansion and substantiation when deployed to relevant land system research tasks. In closing, the directions and key themes for further development of people–place interactions in land system science are discussed.

## Why place?

Out of the numerous challenges currently connected to achieving sustainable development, most can be associated with population growth and urbanisation (Crutzen 2002; Zalasiewicz et al. 2011), which in turn are responsible for sprawl and landscape degradation on an ever accelerating scale (Meeus and Gulinck 2008; Seto et al. 2013; La Rosa et al. 2018). These challenges are perhaps best illustrated by peri-urbanisation, which is the transformation of rural and natural areas into landscapes that are neither urban nor rural and which “may be the dominant urban form and spatial planning challenge of the twenty-first century” (Ravetz et al. 2013). Negative consequences of peri-urban areas include environmental as well as socio-cultural aspects (Nilsson et al. 2013), but most importantly, they present us with increasingly complex landscape configurations and demands placed upon them (Verhagen et al. 2018). Land system science (Verburg et al. 2013; Wu 2019) has made advancements in explaining, quantifying and predicting changes of such complex landscapes, yet landscape degradation and sprawl continues with no signs of slowing down (Seto et al. 2012; Zasada et al. 2013; Verburg et al. 2015).

Further advancements of the land system science research agenda have been put forward to tackle these negative aspects and address critical knowledge gaps (Verburg et al. 2013, 2015), such as the representation and inclusion of human behaviour in land use change modelling (Filatova et al. 2013; Arneth et al. 2014; Verburg et al. 2016; Schlüter et al. 2017), the ability to make nuanced and evaluative judgements on measurements and predictions (Nielsen et al. 2019) and most importantly the call for the land system community to transform its knowledge into practicable solutions (Childers et al. 2015; Verburg et al. 2015; Nielsen et al. 2019; United Nations 2019).

Integrating the concept of place has been proposed as a way of bridging the above knowledge gaps and as an essential aspect of landscape change (Burgess 1979; Buchecker et al. 2003; Hunziker et al. 2007; Williams 2014; von Wirth et al. 2016; Raymond et al. 2017; Kienast et al. 2018). The inclusion of specific local contexts has been advocated and practiced within land system science through place-based research (Balvanera et al. 2017; Masterson et al. 2017). This type of research has shown that including place can make landscape degradation more visible, help in understanding the role of behaviour and motivation in place change, or show how local qualities affect people's stewardship of their landscapes (Williams et al. 1992; Buchecker et al. 2003; Hunziker et al. 2008; Chapin et al. 2010). The degradation of local qualities and lack of effective solutions are particularly evident and challenging in the context of peri-urban areas (Seto et al. 2012, 2013; Geneletti et al. 2017), so that shifting the focus from quantities to qualities is suggested as a way of improving natural and urban landscapes (UN-Habitat 2013; Childers et al. 2015; van Vliet et al. 2019).

Moving beyond place-based research, a more universal conception of place applicable to diverse research tasks is not easily definable and hence remains overlooked (Lewicka 2011). The aim of this paper is to untangle the concept of place and suggest how it can be integrated within land system science. This aim is pursued to better understand the meaning behind the results of socio-ecological system (Ostrom 2009; Stauffacher and Krütli 2016) and land use/land cover change analyses and predictions and, from this understanding, derive better solutions. In other words, we contribute herein our supposition that if place is considered as being more than “a location”, it can be understood as the emergent interaction between people and their environment—thus giving place the potential to be operationalised as a result of socio-ecological systems.

The above-postulated aims are closely aligned with one of the many facets of sustainability science research, which “is seeking to support the integrative task of managing particular places where multiple efforts to meet multiple human needs interact with multiple life-support systems in highly complex and often unexpected ways” (Clark 2007). Particularly in the context of landscape sustainability and its six Es (Musacchio 2009), the aim of this paper is meant to expand our understanding of the aesthetics, experience and ethics when studying or developing socio-ecological systems.

This text is organised into two main sections, with the first section focusing on the past and current state of place research. An overview of place studies is presented in order to form a base for connecting the concepts and recommendations presented in the sections thereafter. We then outline the challenges of place studies by referring to methodologies which incorporate place within land use change research. Out of this follows the second section, which presents a possible future for place in the context of land system science. In this final section, we first propose a description of place and place-making, closing with a discussion of how to apply it to possible research tasks in land system science.

## Place studies: past and present research

**Fig. 1:**
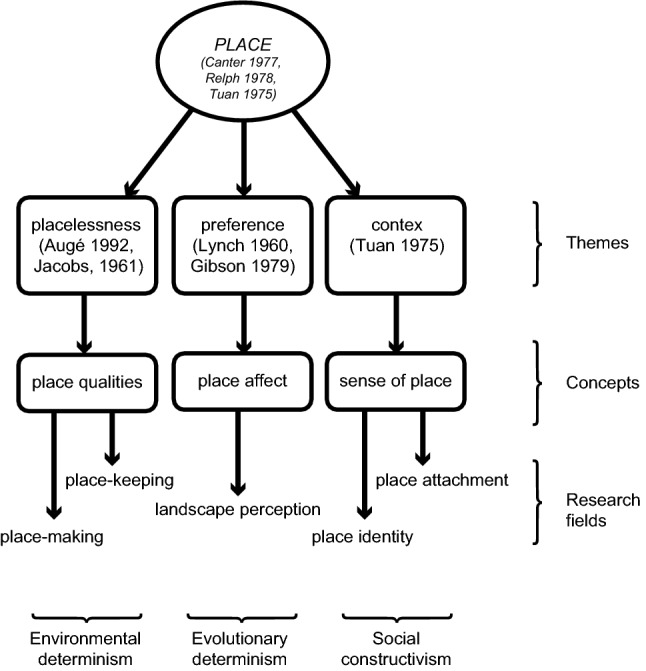
Overview of place studies ordering specific research fields within three thematic positions

The concept of place emerged in the social sciences as an attempt to reframe and capture the complexities of our physical surroundings and our relationship to them (Lewicka 2011; Sepe and Pitt 2014). A wide spectrum of disciplines have confronted themselves with place as a concept, accordingly resulting in a variety of place-related constructs such as sense of place, place identity, place-keeping or place-making (Cresswell 2004; Williams 2014; Gieseking et al. 2014). As a result, there is a possibility for such an all-inclusive concept as place to become vague or difficult to navigate. A clear conceptual base is, therefore, necessary for operationalising place within land system science. We do this by organising place studies in a novel way, which connects the dimensions of place with their respective research fields (Fig. 1).

The principal foundations for place studies were laid out by Canter (1977), Relph (1976) and Tuan (1975) by investigating the interactions between people and their environment (Lewicka 2011; Sepe and Pitt 2014). Although these researchers approached the study of place from different positions, a departure point common to all three were environments described by their authors as being “placeless”. Taking this further, they explicitly formulated that describing and understanding how place is experienced should be used to avoid the creation of such placeless environments (Canter 1977).

**Fig. 2:**
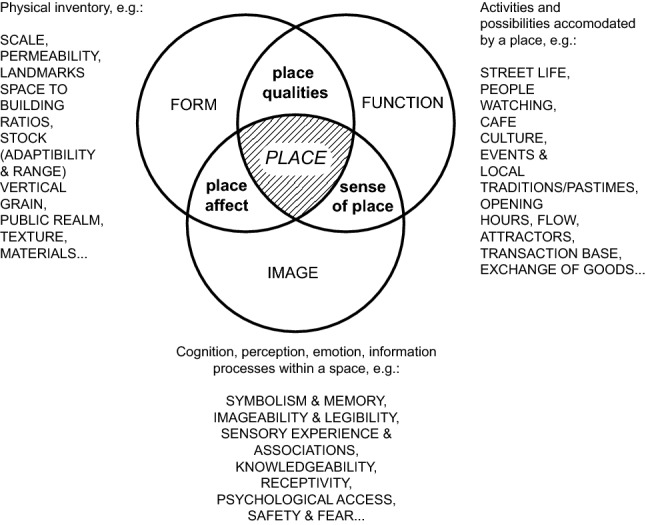
Dimensions of place with examples (adapted from Canter (1977) and Montgomery (1998)) and resulting concepts in bold (place qualities, sense of place, place affect)

The dimensions of place are classified into (i) physical *form*; (ii) observable function or *activity*; (iii) and the experiential meaning or *image* (Figure 2) (Canter 1977; Montgomery 1998). Differing classifications still follow the above categories and are usually just expanded upon or modified by an additional item (Agnew 1987; Pancholi et al. 2017). A common way of arranging the different approaches to place is to classify them as representing environmental determinism or social constructivism (Rapoport 1977; Carmona et al. 2003; Hunziker et al. 2007; Vischer 2008; Dempsey 2009).

*Environmental determinism* postulates that physical features (form) take the primary role in how people experience space around them. It refers back to *placelessness* in a principal way (Jacobs 1961; Augé 1992), arriving at *place qualities* as the determining factor in the causal relationships between environment and behaviour (Alexander et al. 1977; Montgomery 1998). This branch of place studies engenders the concepts of *place-making* and *place-keeping*. Although place-making can be used to refer to a number of different ideas, in this branch of place studies, it is interchangeably used with term “human-centred design”, which essentially means the provision of public spaces and mitigating the negative effects of motorised traffic (Beske 2018).

An equally important branch of place studies, *social constructivism* takes context as its point of departure and investigates the *sense of place* in a causal way (Tuan 1975; Hummon 1992). Social constructivism stands in contrast to environmental determinism, stating that it is personal experience (function) and the therein resulting meanings (image), which primarily shape our relationship to space. Out of this approach emerge the concepts of *place identity* and *place attachment* (Low and Altman 1992). Place attachment focuses on the emotional bonds between people and specific places, usually studied across longer time spans (as opposed to immediate reactions).

The above-postulated dichotomy can be supplemented by a third, and altogether different area of place studies. Although it is often categorised as environmental determinism (Hunziker et al. 2007), our place studies framework refers to this area as *psychosensory universalism*. This strand of research focuses on the interactions between form and image, seeking to understand commonly felt *preferences* for landscapes and can be described as studying *place-affect*. In understanding the preferences for elements and their arrangement (traditionally within natural landscapes), place-affect is interested in the immediate and universal reaction to a place, by investigating the mechanisms and the role of affect and cognition in relation to perception. The most tested theories, which fall into this segment of place studies, are the psycho-evolutionary model (Ulrich 1983) and the information processing theory (Kaplan 1987; Hunziker et al. 2007).

To summarise thus far, place studies constitute a still developing area of interest and stem a variety of fields investigating particular aspects or interaction found to occur in places (Fig. 2). Environmental determinism developed place-making by focusing on *place qualities* through the interaction of form and function (i.e. activities). Social constructivism helped to shape place-attachment and investigated *sense of place* through the interaction of function and image. Psychosensory universalism incorporates *place affect*, through research understanding landscape preference primarily as an engrained neuro-physical reaction and focuses on the processes between form and image. Although a wide range of indicators have been used for particular place dimensions or interactions, specific sets of indicators to describe place as a totality have been suggested by architects and urbanists (Montgomery 1998; Ewing et al. 2013) but remain to be systematically applied and validated.

There is an emergent trend of a more inclusive treatment of people–place relationships by developing approaches that acknowledge all three dimensions of place as being integral to place studies (Koskela 2008; Vischer 2008; Dempsey 2009; von Wirth et al. 2016; Raymond et al. 2017). Examples illustrating interactions between all three dimensions of place (form, function, image) can be found in engineering disciplines attempting to include qualitative features in a quantitative framework. Illustrating this approach are recent studies on walkability, which show that including factors besides form (e.g. block size) or function (e.g. trip purpose), but also image (e.g. safety) can lead to more complete model predictions (Lai and Kontokosta 2018).

The most fundamental challenge for place study research is to translate the various definitional questions into mechanisms and causalities behind humans’ relationships to place (Lewicka 2011). Some research methods attempt to nevertheless overcome this challenge through novel approaches. An explorative overview of the different treatments of place implemented within research relevant to land system science is presented in Table 1. Rather than attempting to define the single most appropriate method, the aim here is to draw up a spectrum of how place could be operationalised.

**Table 1:** Examples of the treatment of place within research relevant to land system science

Project	Method	Aim	Dimensions of place	Landscape type
*Place as process*				
Pancholi et al. (2018)	Stakeholder focus interviews	Identifying role of social groups in influencing place-making	Function, image	Peri-urban commercial
Loepfe (2014)	Stakeholder interviews, qualitative case study comparisons, actor network mapping	Planning and political dynamics influencing place-making	Form, function	Peri-urban settlements and centres
Lewicka (2005)	Survey, structural equation modelling	Effect of social and cultural factors on place attachment	Function, image	Urban residential
Arefi (2014)	Historical and architectural analysis of case studies	Guiding principles behind neighbourhood development	Form, function, image	Urban neighbourhoods
*Place as product*				
Jonietz (2016)	Agent-based modelling	Modelling subjective micro-walkability	Form, function	Urban centre
Ewing et al. (2013)	Field observations	Describing the amount of various elements to compare places	Form, function	Urban centre
March et al. (2012)	Field observations, factor analysis	Measuring building adaptability and street vitality	Form, function	Urban centre
Porta and Renne (2005)	Field observations	Deriving formal indicators for urban sustainability	Form, function	Urban neighbourhoods
*Place as product and process*				
Canter and Rees (1982)	Survey, factor analysis	Elements of residential satisfaction	Form, function, image	Suburban residential
Del Aguila et al. (2019)	Survey, factor analysis	Public space meaning and behaviour	Form, function, image	Urban centre
Hillier and Hanson (1984)	Space syntax	Defining spatial metrics of urban form and their influence on social behaviour	Form (function, image)	Urban neighbourhoods
Lindal & Hartig (2013)	Computer-generated visualisations, structural equation modelling	Restorative qualities of architectural elements in residential streetscapes	Form, image	Suburban residential
Singh et al. (2008)	Photographic landscape representations, structural equation modelling	Expanding landscape preference model (Kaplan 1987) to include cognitive and behavioural components	Form, image	Rural, natural landscape
Pérez-Soba et al. (2018)	Visioning workshops, scenario development	Establishing visions for sustainable land use in the European Union using cross-sectorial stakeholder engagement	Form, function, image	Urban, peri-urban, rural and natural landscapes

The main observation gained from this overview is that some research focuses on place as an end *product*, while others study the dynamic relationships of people to place as a *process*. Research which approaches place as either only product or process is limited in its applicability when used to derive mechanisms of people–place relationships. There have been attempts to link the interactions between place as both process and product (Table 1), but not without their own set of challenges.

One such challenge is associated with methodological limitations of a particular approach to capture a sufficient range of place interaction types. Canter and Rees (1982) offer perhaps the most complete structure of interactions between personal attitudes (image), environmental attributes (form) and social interaction (function). Although they offer a methodology for inferring causalities between elements from all three dimensions of place, the psychometric scales used therein are not intended to be operationalised in a spatially explicit way. This approach thus lacks the transferability to land system science and would require further modification before it could be used, for example, as part of a land change model. On the other hand, space syntax (Hillier and Hanson 1984) offers a set of spatial metrics (form) as dependent variables of place, but due to its flexible framework requires systematic research to discern how these metrics influence the other place dimensions (function and image). In addition, space syntax was developed to study urban environments and would perhaps need further adaptation for more rural and natural places found within the rural–urban continuum.

Some approaches do not study all dimensions of place due to their inherent research aims. Although examples such as Singh et al. (2008) recognise place as a product and process, they purposely leave out function to better understand the interaction between image and form in their research on affective processing strategies. Lastly, a promising way to approach the topic is to use stakeholder engagement to produce concrete descriptions of place and help guide discussion of the necessary or possible processes to reach such place visions (Pérez-Soba et al. 2018). However, the qualitative and context-specific nature of these participatory approaches would require more stringent structuring (thus working against their intended adaptability) and a sufficiently large sample size to infer underlying dynamics of place within land use change.

To recapitulate the challenges of incorporating place within land system science, we consider that (1) the understanding of place as both product and process is paramount; (2) there seems no single methodology suited to operationalising place, implying the necessity of complementary or hybrid approaches; (3) the connection between qualitative or normative components with quantifiable spatial metrics remains to be solved in operationalising place.

## Describing place and place-making for land system science

In the following section, we attempt to provide a definition of place based on the previously described research and we introduce place-making to support the operationalisation of place in land system science.

**Fig. 3:**
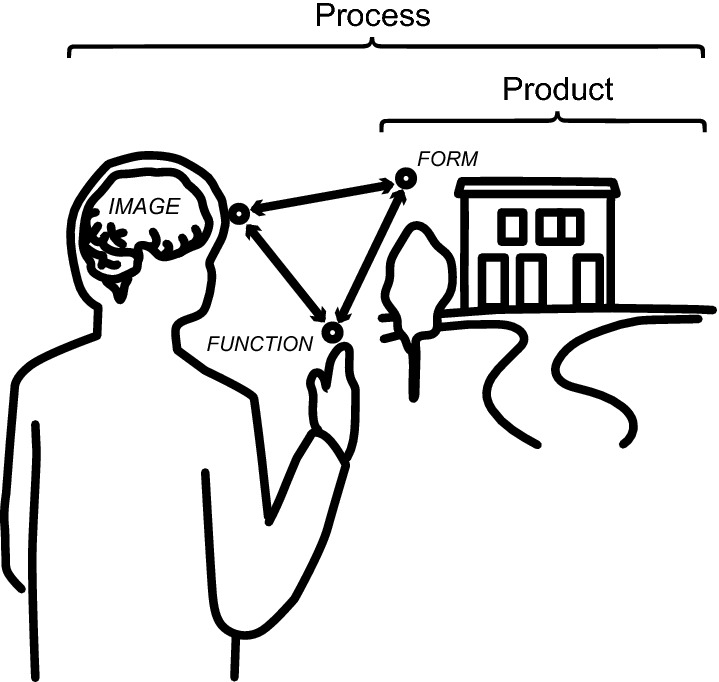
Distinguishing the concepts of place as a product (“a place”) and dynamic experiential process

*Place is physical space as experienced by a person.* By incorporating experience (understood here as a dynamic interaction between a person and their environment), place can potentially be discussed interchangeably as both a product (“a place”) and an experiential process (Fig. 3). Considered as a product, place allows for truly any classifications and components (for example dream space or public space). However, place in the context of a particular process can only include components, which are explicitly connected in a causal relationship with each other and are relevant to that process. The need for clarity in distinguishing these facets of place (product and process) becomes apparent.

*Place-making is the set of processes, which generate and change places.* The term place-making is introduced to more clearly constrain place in the form of a causally connected set of components relevant to how places change. In other words, as opposed to place considered on its own, place considered in tandem with place-making becomes a more concrete concept and can potentially circumvent many definitional issues. Consequently, the term allows for a more explicit differentiation of product (place) from process (place-making), when discussing these concepts in a research context (Fig. 3). The above definition of place-making allows for both physical creation of spaces, as well as the very experience of a physical space itself in generating places. In other words, building a highway (physical place-making) and fostering social networks with neighbours (non-physical place-making) are two extremes which both fall into the above definition of place-making. However, not all types of place-making are equally relevant to land use change. This important distinction can be used to further constrain the set of possible types of place-making, which need to be addressed in the context of land system science.

**Fig. 4:**
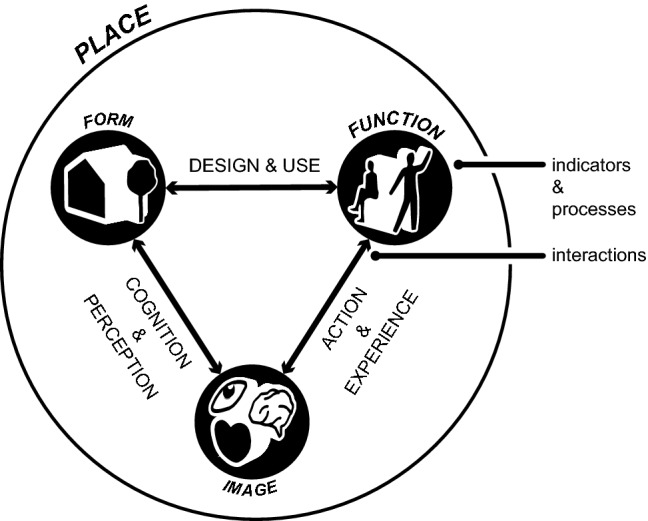
Place as totality of indicators, processes and interactions organised into three dimensions

Our proposed model of place for land system science includes three dimensions (Fig. 4): form (physical characteristics and inventory of a place), function (possible and actual activities accommodated by a place) and image (how a space is understood or perceived based on affective-cognitive processes). These three dimensions can be further described by their own internal processes (e.g. the role of past experiences on our image of a place, or the amount of people dictated by a particular activity), as well as specific indicators (e.g. safety, mood or memory, in the case of image). These dimensions are connected with each other through various interactions, for example the influence of density (form) on our perception of safety (image) and business opening times (function). Through this conceptualisation, we further refine the previous description and propose that *place is the totality of components, processes and interactions necessary to describe the experience of physical spaces* (Fig. 4). Considered this way, this final description of place allows for case-specific discussions relevant to different research contexts within land use science. With the help of the above conceptualisation, there is a possibility of defining “ecological places”, “urban places” or even “urban-ecological” places (Childers et al. 2015). Communicating the kind of place one might experience contrasts here with a universalist conception of place, which requires definition through a specific intersecting set of yet to be discovered components (Fig. 2) to judge whether a location is a place or not.

Based on the insights gained from our exploration of place studies, we find our proposed definition of place to be the most elementary structure, which nevertheless allows further expansion or elaboration for specific research contexts. For example, the role of governance type cannot be neatly assigned to any of the three dimensions of place. Instead, the influence of governance on place can be captured through its impact on any of the main dimensions (e.g. impact on function through banning of certain activities); in other words, by being included within the internal processes of a specific dimension.

**Fig. 5:**
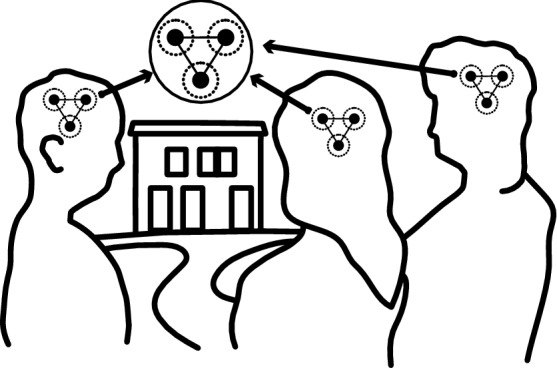
Place as an emergent process within land system science

Most importantly perhaps, our way of conceptualising place can potentially be applied to emergent phenomena (Goldstein 1999). Each of the dimensions can be defined across a range of people and places, where ultimately the sum of interactions can be used to define a place (Fig. 5). This point shows the importance of distinguishing product from process in the context of place. It also shows how place-making can be operationalised by directly using the dimensions of place, transposing them onto agents within an environment and adding a temporal component to the interactions and processes.

The final observation to be made is that current research aims within the land system community (GLP 2019) can be aligned with the above-postulated description of place. Incorporating place within land system research allows to focus on more subtle land use changes, it requires the inclusion of individual and social behaviour in socio-ecological interactions and the consideration of dynamic feedbacks between the different dimensions and their components (Verburg et al. 2015).

## Operationalising place(-making)

Considering the previously outlined state of research and methodologies of place studies in combination with the above description of place allows for a number of recommendations to be made for land system science.

### Complexity and complementarity

Suitable research contexts have to be chosen, where the operationalisation of place constitutes an added value in terms of advancing current methodologies. Complex landscapes, such as peri-urban areas within the rural–urban continuum, offer such contexts and have for example posed challenges in defining and monitoring of these landscapes (Siedentop and Fina 2010; Zasada et al. 2013; van Vliet et al. 2019).

A plurality of research methods integrated within a single complementary model is a necessity for place research. The differences in interaction types (e.g. form and function versus form and image) require by their nature very different research methodologies. To illustrate, this might involve cognitive psychology connected with sociology and economy within an iterative research approach. Consequently, it is clear that specialists from disciplines outside of land use and landscape science or land use policy, to name a few, are needed within the land system science community (Verburg et al. 2013). Integrating behaviour within land use modelling and transforming knowledge into solutions (Verburg et al. 2015) would require the experience of psychologists and architects in closing such gaps with the help of place in land system science.

An example illustrating this very point is a study by Hackman et al. (2019), which uses virtual reality simulations to study the effect of affluent versus disadvantaged neighbourhoods on physiological and emotional activity. When representing places as well as their behavioural triggers in an experimental setup, the various experimental variables become a challenge to account for in a systematic way. Hackman et al., coordinate expert knowledge in a way where each field can contribute their relevant conception of place: architects contribute the physical dimensions and layout of street blocks, social psychologists determine the type of personal interactions in each environment, and game designers provide the form and components that allows adequate immersion for the test subjects. The result is an idealised version of places in order to allow for policy-relevant discussions of neighbourhood development, without losing the required “place-fidelity” of actual neighbourhoods.

### Transformation pathways

The role of transformation pathways within place needs to be investigated, which could form an intermediate step in understanding the more complex concept of place-making. The discussions surrounding placelessness describe a universal transition from desirable places as a product of the past to less valuable modern environments (Relph 1976; Augé 1992). However, the narrative of placelessness is not as straightforward as it seems, since the increasing number of non-places seems to positively influence the interest and importance of place (Lewicka 2011).

One of the many purposes of a detailed investigation into the transformation pathways of places is to gain a better understanding of the role of inherent qualities of places versus the role of time-dependent factors in shaping our reception of places. This understanding would allow to answer whether place-making can be “designed-in” through an appropriate selection of initial place conditions (Dempsey 2008).

One possible approach is to collect insights from places of different ages studied at the same instant (cross-sectional measurements), complemented by the study of the same places measured at different time steps (longitudinal measurements), as demonstrated by Von Wirth et al. (2016). Systematic place monitoring allowed to study the time-dependent dimensions of place attachment and responses to place change in a peri-urban settlement, showing for example that even significant place transformations do not disrupt people’s place bonds, as long as the transformation produces a positive association (von Wirth et al. 2016).

### Emergent properties

Taking the position that place can be best described and understood indirectly as an emergent phenomenon, linking qualities of places with quantifiable spatial metrics is likely to play a major role in place research.

The first step in establishing such links is to determine which indicators are necessary within the three dimensions of place, with the set of possible place metrics being rather expansive (Porta and Renne 2005; Dempsey 2009; Boeing 2018). Since many indicators are expected to be correlated (e.g. number of business types and street life), the selection process would centre around the systematic reduction of indicators until a robust set is found. Architects or environmental psychologist have a working knowledge that allows them to describe, classify and connect place qualities with high acuity (Rapoport 1977; Gehl 2010). Formalising experiential knowledge could thus be the method to operationalise such place indicators. This corresponds to place as something learned and which can be used as an immediate source of information (Raymond et al. 2017). A detailed understanding of how this occurs can, therefore, potentially increase our understanding of how qualities define places in more absolute terms. Salesses et al. (2013) could show that this experiential knowledge can be elicited through crowd-sourced information and combined with deep learning to allow for the classification for a set of perceptual qualities (safety, social class, uniqueness), delivering stable results in places sourced from various cities.

### Multi-scale place-making

The choice of scale defines how place can or should be represented—and as pointed out previously, almost anything can be defined as a place, but not all definitions are relevant to land use change. This could potentially be helpful in reducing the number of components and interactions that need to be represented. A cursory glance at place studies would allow to conclude that issues of preference, norms and perception are too subjective, temporally variable and dynamic, to allow for systematic study. Similar difficulties are found and overcome in the study of travel behaviour, where individual processes are derived from large aggregates by, for example, combining personal travel surveys (model indicators) with traffic count data (validation and calibration) (Ortúzar and de Willumsen 2011).

In the case of place within land system science applications, more than one scale needs to be integrated and their reciprocity studied (Wissen Hayek et al. 2015). Individual behaviour will need to be inferred from a large aggregate (e.g. neighbourhood level) and vice versa: the effect on individuals due to changes occurring on a higher scale has to be captured. This aligns with our postulate that place and place-making can be thought of as an emergent phenomenon (Fig. 5), as “the construct of emergence is appealed to when the dynamics of a system seem better understood by focusing on across-system organization rather than on the parts or properties of parts alone.” (Goldstein 1999).

Novel approaches, which allow the integration between scales, are thus going to be a necessity to negotiate their associated processes when assessing place and place-making. A possible approach to find a balance between multiple scales and model complexity (and its associated bottlenecks such as processing power or interpretability) is by incorporating methodologies such as agent functional types, which are intended to allow modelling of actor behaviour in a scalable way to cover larger spatial extents, while representing local conditions (Rounsevell et al. 2012; Arneth et al. 2014; Murray-Rust et al. 2014).

## Conclusions

In bringing the previous points together, we postulate that a better understanding of place for land system science can be gained through the study of complex landscapes at multiple scales, by attempting to connect qualities with quantities through complementary methodologies. Although place is not universally implemented within land system science, it is not completely foreign or incompatible as a concept either. Among a plurality of positions, there is no single correct concept of place, but rather a pool of knowledge to draw from.

Therefore, we propose that place is best operationalised as a multi-level concept, which includes dynamic interactions between various spatially relevant dimensions and their components (Fig. 4). Consequently, place has social, individual and environmental components, requiring contrasting approaches from different research disciplines, all the while being unified within a single conceptual model. Land system science lends itself to this challenge particularly well, especially considering recent calls for further development of its research agenda (Verburg et al. 2015). In other words, land system science can potentially help us understand place better; and place can help further our understanding of complex land systems.

However, as evident throughout this text, we are finding ourselves at the early stages of understanding and using place within the contexts of land use change and sustainability challenges. Aspects such as definitional issues, the need to collect new datasets on place qualities or consolidating and innovating on existing land system science methodologies (e.g. refining behaviour and decision-making within agent-based models to include place-making dimensions), are some of the challenges likely to be encountered moving forward.

A possible path ahead would be to first demonstrate how places can be defined within different complex socio-ecological systems and analyse what processes a given place-definition would allow to capture. How relevant these processes are for land use change and their contribution to sustainable development could become the deciding factor in how place should ultimately be operationalised. Whatever the route ahead might be, the key in operationalising place is to iteratively “connect the dots”: connecting theory to experiments, data collection with descriptive analyses or land use change with environmental psychology. The takeaway message is that a valuable body of knowledge in the field already exists and although at first its wealth will be challenging to parse, it is this very wealth that makes the applied pursuit of place such a worthwhile endeavour.
